# Insulin-like growth factor-1 (IGF-1) induces the activation/phosphorylation of Akt kinase and cAMP response element-binding protein (CREB) by activating different signaling pathways in PC12 cells

**DOI:** 10.1186/1471-2202-7-51

**Published:** 2006-06-22

**Authors:** Wen-Hua Zheng, Rémi Quirion

**Affiliations:** 1 Department of Pharmacology, School of Pharmaceutical Sciences, Sun Yat-sen University, Guangzhou, China; 2 Douglas Hospital Research Center, Department of Psychiatry, McGill University, Montreal, Quebec, H4H 1R3, Canada

## Abstract

**Background:**

Insulin-like growth factor-1 (IGF-1) is a polypeptide growth factor with a variety of functions in both neuronal and non-neuronal cells. IGF-1 plays anti-apoptotic and other functions by activating multiple signaling pathways including Akt kinase, a serine/threonine kinase essential for cell survival. The nuclear transcription factor cAMP response element-binding protein (CREB) may also be involved although relationships between these two proteins in IGF-1 receptor signaling and protection is not clear, especially in neuronal cells.

**Results:**

IGF-1, in a concentration- and time-dependent manner, induces the activation/phosphorylation of Akt and CREB in PC12 cells by activating different signaling pathways. IGF-1 induced a sustained phosphorylation of Akt while only a transient one was seen for CREB. The phosphorylation of Akt is mediated by the PI3 kinase pathway while that of CREB is dependent on the activation of both MAPK kinase and p38 MAPK. Moreover, the stimulation of PKC attenuated the phosphorylation of Akt induced by IGF-1 while enhancing that of CREB. Survival assays with various kinase inhibitors suggested that the activation/phosphorylation of both Akt and CREB contributes to IGF-1 mediated cell survival in PC12 cells.

**Conclusion:**

These data suggest that IGF-1 induced the activation of Akt and CREB using distinct pathways in PC12 cells.

## Background

Insulin-like growth factor-1 (IGF-1) is a polypeptide trophic factor playing important roles in the survival and differentiation of both neuronal and non-neuronal cells [1,2]. The biological actions of IGF-1 are mediated by a heterotetrameric tyrosine kinase receptor, the IGF-1 receptor, which is similar to the insulin receptor both in structure and functions [2,3]. Binding of IGF-1 to its receptor causes receptor autophosphorylation and the activation of intrinsic tyrosine kinase. Activated receptor kinase phosphorylates various intracellular proteins like the insulin receptor substrate-1 (IRS-1) and Shc [3-5], leading to the activation of multiple signaling pathways including the phosphatidylinositide 3 kinase (PI3K)/Akt pathways and the mitogen-activated protein (MAP) kinase (also called extracellular signal-regulated kinase; ERK; [2,3,6,7]).

Akt, a key target of the PI3 kinase, is a serine/threonine kinase that plays critical roles in the modulation of cell development, growth and survival [8-10]. Stimulation of cells with IGF-1 induces the activation of PI3 kinase leading to increased levels of phosphatidylinositol [3,4] diphosphate [PI [3,4] P2] and phosphatidylinositol [3-5] triphosphate [PI [3-5] P3] in target cells [11,12]. This event recruits Akt to the plasma membrane where it is phosphorylated by PI [3-5] P3 dependent kinase, (PDK)-1 and -2, respectively at residues Thr308 and Ser473 [13,14]. The phosphorylation of these residues activates Akt kinase which can then phosphorylate its many substrates including glycogen synthase kinase-3 (GSK-3) [15], the Bcl-2 family member Bad [16], caspase-9 [17], nuclear factor-κB (NFκB) [18,19] and the winged-helix family of transcription factors, FOXO1, FOXO3a and FOXO4 [1,10,20-22], leading to cell survival and the inhibition of apoptosis [1,8,10,23].

The Ca^2+^/cyclic AMP response element-binding protein (CREB) is one of the common nuclear targets of tyrosine kinase receptors playing important roles in many biological functions including neuronal plasticity, full axonal development, memory consolidation, and neuroprotection [24-30]. This transcriptional factor belongs to the CREB/ATF family and binds to the specific sequence, 5'-TGACGTCA-3' known as CRE [31]. Activation of this transcription factor requires the phosphorylation of the Ser-133 residue which increases its association with CREB-binding protein [32]. Several kinases including cyclic AMP-dependent protein kinase (PKA), protein kinase-C (PKC), calcium/calmodulin-dependent protein kinases, MAPK/p38 MAPK/MAPKAP kinase-2, ribosomal S6 kinase (RSK) family of kinases, the mitogen and stress-activated protein kinases 1 (MSK1) and Akt have been shown to be capable of phosphorylating this protein on Ser-133 residue [28,33-38].

IGF-I stimulates the phosphorylation of CREB and regulates the expression of a number of CRE-containing genes including bcl-2 and c-fos in several cell types [32,39]. Interestingly, CREB is reported as a possible target of Akt [30,37] suggesting that it may be a target of Akt in IGF-1 mediated survival. However, reports about Akt as a CREB kinase in IGF-1 signalling is still rather controversial with one report suggesting that the phosphorylation of CREB induced by IGF-1 is independent on Akt [40]. Moreover, the signalling of CREB and Akt is cell type-dependent and effectors specific [41]. Therefore, it is deemed important to clarify the role of Akt in the phosphorylation of CREB induced by IGF-1. Accordingly, we characterized here the signalling of IGF-1 stimulated activation of CREB compared to that of the PI3K/Akt in PC12 cells. Our data show that IGF-1 promotes the phosphorylation of Akt and CREB in these cells. The activation of Akt is mainly mediated by the PI3 kinase pathway, while that of CREB is primarily dependent on the activation of MAPK and p38-MAP kinases revealing the differential regulation of these two proteins by IGF-1 receptor signalling. It also argues against a key role for Akt as a CREB kinase in PC12 cells. The survival study suggests that the activation of these two proteins likely contributes to the survival effects of IGF-1 in PC12 cells, with the PI3K/Akt kinase pathway playing a predominant role.

## Results

### IGF-1 stimulates the phosphorylation of Akt and CREB in PC12 cells

**Figure 1:**
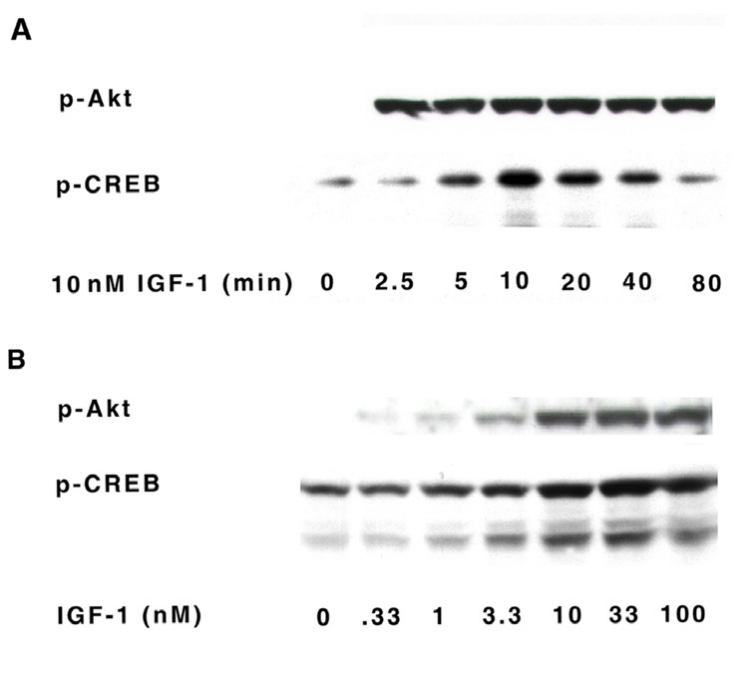
**IGF-1 induced time- and concentration-dependent phosphorylation of Akt and CREB in PC12 cells**. PC12 cells were treated with 10 nM IGF-1 for various times and then the phosphorylation of Akt and CREB were determined as described in Methods. **A). Time-dependence and (B). Concentration-dependence. **Blots represent prototypical examples of experiments replicated at least 3 times.

To investigate the effect of IGF-1 on the activation/phosphorylation of Akt and CREB in neuronal cells, PC12 cells were treated with 1–100 nM IGF-1 and the phosphorylation of Akt and CREB evaluated as described in Methods. Figure 1 shows that IGF-1 induced the sustained phosphorylation of Akt while a transient phosphorylation was seen for CREB. In the case of CREB, one additional band with a lower molecular weight was seen in the blot. This band represents p-ATF which has 100% homologous consensus phosphorylation sequence with CREB and cross react with the anti-pCREB antibody [30]. Treatment of PC12 cells with 10 nM IGF-1 caused a 3–5 fold increase in the phosphorylation of Akt at Ser-473. The phosphorylation reached the highest level at 2.5 min and remained unchanged for over 40 min. The phosphorylation of CREB at Ser-133 was increased 2–3 fold by 10 nM IGF-1. The induction of CREB phosphorylation was evident at 5 min, peaked at about 10 min and decreased thereafter (Fig 1A). IGF-1 also concentration-dependently stimulated the phosphorylation of Akt and CREB in PC12 cells. The effect of IGF-1 on Akt was seen at concentration as low as 0.33 nM while about 3 nM was required to induce the phosphorylation of CREB (Fig 1B).

### The phosphorylation of Akt by IGF-1 is mediated by PI3 kinase while MAPK and p38 MAPK regulate IGF-1 induced phosphorylation of CREB

**Figure 2:**
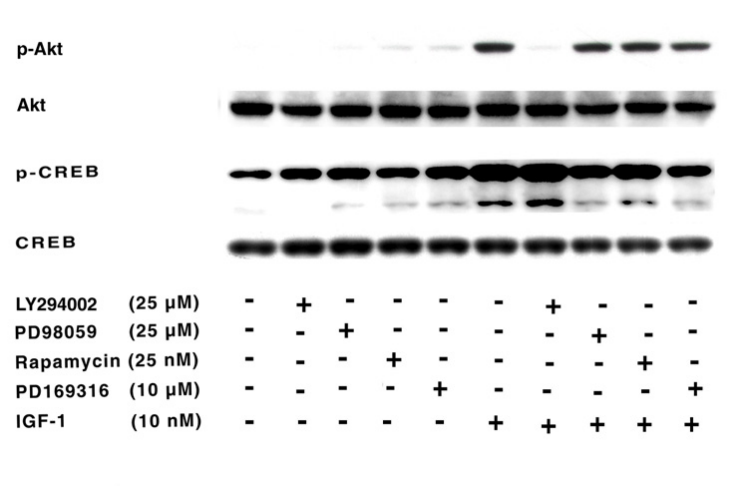
**IGF-1 induced the phosphorylation of Akt in PC12 cells via the PI3 kinase pathway while MAPK and p38 MAPK pathways mediated the activation of CREB induced by IGF-1**. Following treatment with different kinase inhibitors for 40 min, PC12 cells were exposed to 10 nM IGF-1 and the phosphorylation of Akt and CREB was determined by Western blots using anti-phospho- Akt/CREB antibodies. The PI3 kinase inhibitor LY294002 inhibited IGF-1-induced phosphorylation of Akt in PC12 cells while a MAPK pathway inhibitor PD98059, a p38 MAPK inhibitor PD169316 and a p70S6 pathway inhibitor rapamycin, did not have any significant effect. In contrast, the phosphorylation of CREB induced by IGF-1 was partially blocked by inhibitors of both MEK (PD98059) and p38 MAP kinase (PD169316). Blots represent prototypical example of experiments replicated at least 3 times.

**Figure 3:**
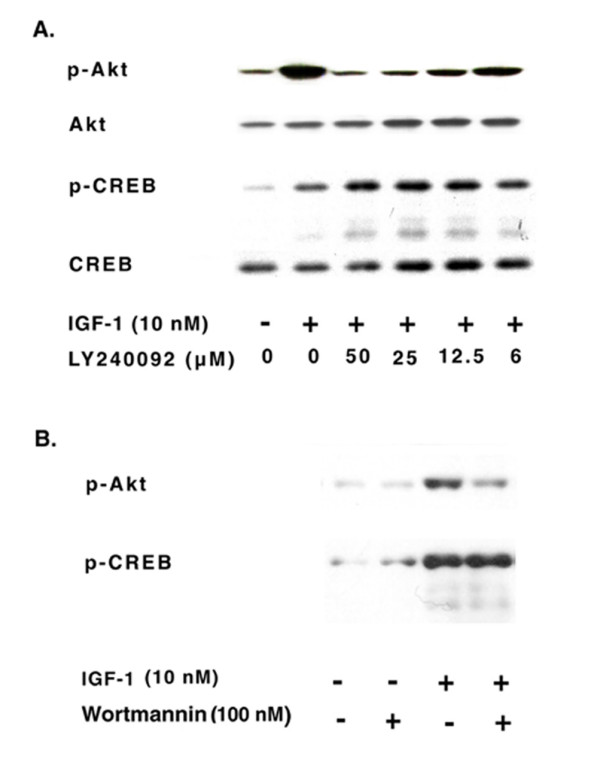
**PI3 kinase inhibitors blocked the activation of Akt by IGF-1 while has no effect on the phosphorylation of CREB**. PC12 cells pretreated with different concentrations of LY294002 or 100 nM wortmannin were stimulated with 10 nM IGF-1 and the phosphorylation of Akt and CREB was determined. (A) LY294002 blocked IGF-1-induced phosphorylation of Akt in a concentration-dependent manner while slightly enhancing the activation of CREB in PC12 cells. (B) 100 nM wortmannin significantly blocked the activation of Akt while having no effect on CREB phosphorylation. Blots represent prototypical example of experiments replicated at least 3 times.

Having established that IGF-1 can induce the phosphorylation of Akt and CREB, we studied next the signaling pathways mediating the action of IGF-1. PC12 cells were pretreated with various kinase inhibitors before adding IGF-1. Figure 2 (lane 6 versus 1) demonstrates that 10 nM IGF-1 causes a 3–6 fold increase in the phosphorylation of Akt. Pre-treatment with the PI3 kinase inhibitor, LY294002 (25 μM), blocked IGF-1 induced activation of Akt while slightly enhancing the phosphorylation of CREB (Fig 2A, lane 7 vs 6). In contrast, the MEK inhibitor PD98059 (25 μM; Fig 2, lane 8 vs 6), the p70 S6 kinase pathway inhibitor rapamycin (25 nM, Fig 2, lane 9 vs 6), and the p38 MAPK kinase inhibitor PD169316 (10 μM, Fig 2, lane 10 vs 6) failed to significantly alter IGF-1-induced Akt phosphorylation while partially but significantly attenuating that of CREB. Additional experiments revealed that the inhibitory effect of LY294002 on IGF-1-induced Akt phosphorylation was concentration-dependent (Fig. 3A) with a maximal effect observed at 50 μM.

To extend these results further, wortmannin, another well known PI3 kinase inhibitor, was investigated in our model. Wortmannin (100 nM) had no effect on IGF-1 stimulated phosphorylation of CREB but most significantly blocked that of Akt demonstrating further the differential mechanisms used by IGF-1 to regulate their phosphorylation (Figure 3B).

### MAPK kinase and p38 MAP kinase inhibitors concentration-dependently inhibit IGF-1-induced phosphorylation of CREB

**Figure 4:**
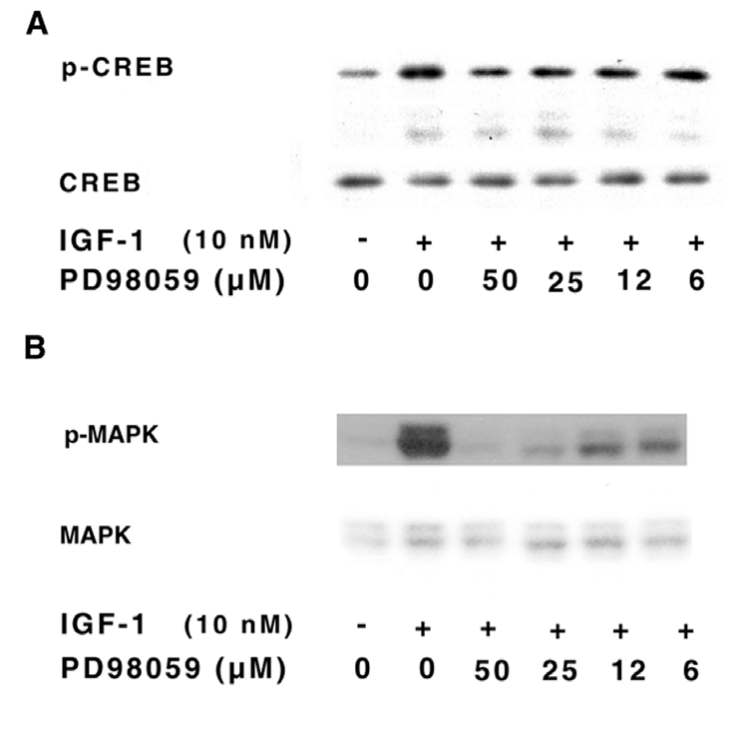
**The MEK kinase inhibitor PD98059 attenuated IGF-1-induced activation of MAPK kinase and CREB in PC12 cells**. PC12 cells pretreated with various concentrations of the MAPK pathway inhibitor PD98059 were incubated with 10 nM IGF-1 for 10 min. Cells were collected and the activation of MAPK and CREB were determined as described in Methods. PD98059 concentration-dependently blocked the activation of MAPK stimulated by IGF-1. This inhibitor also attenuated the phosphorylation of CREB in a similar manner. Blots represent prototypical example of experiments replicated at least 3 times.

**Figure 5:**
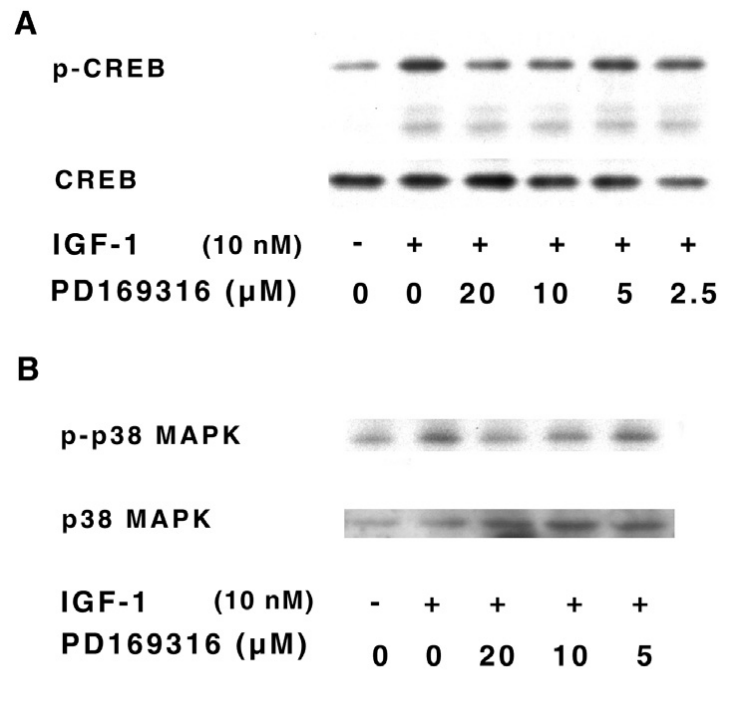
**The p38 MAP kinase is involved in the phosphorylation of CREB induced by IGF-1 in PC12 cells**. PC12 cells were treated with the p38 MAPK inhibitor PD169316 (2.5–20 μM) and 10 nM IGF-1, and the phosphorylation of CREB and p38 MAPK were measured by Western blot with anti-phospho-CREB and phospho-p38MARK antibodies. PD 169316 significantly blocked the phosphorylation of p38 MAPK and CREB induced by IGF-1. Blots represent prototypical example of experiments replicated at least 3 times.

To investigate in detail the role of MAPK and p38 MAPK kinases on the phosphorylation of CREB, well established inhibitors of these two pathways were used. As shown in Figure 4, the MAPK pathway inhibitor, PD98059, concentration-dependently inhibited IGF-1 stimulated phosphorylation of CREB (Fig 4A) and MAP kinase (Fig 4B). Similarly, Figure 5 shows that the phosphorylation of p38 MAPK and CREB is concentration-dependently inhibited by a p38 MAPK specific inhibitor, PD169316.

### PMA attenuates IGF-1-induced phosphorylation of Akt while increasing CREB phosphorylation

**Figure 6:**
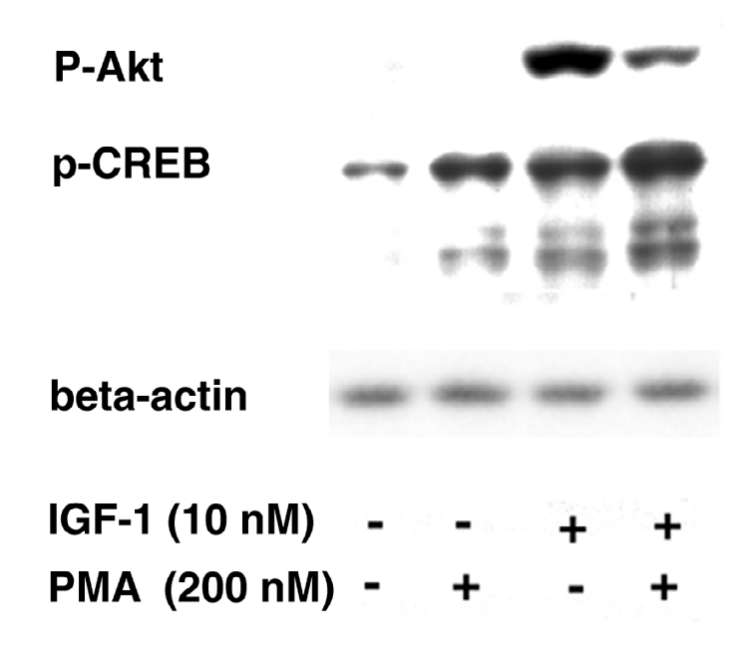
**PMA attenuated the phosphorylation of IGF-1-induced activation of Akt while enhancing the phosphorylation of CREB**. PC12 cells were treated with 400 nM PMA before incubation with 10 nM IGF-1 and the phosphorylation of Akt and CREB determined. PMA treatment decreased the phosphorylation of Akt while increasing the activation of CREB. Blots represent prototypical example of experiments replicated at least 3 times.

We have previously shown that the activation of PKC by PMA attenuated the phosphorylation of Akt induced by IGF-1 [42]. We explored here the effects of such a treatment on Akt versus CREB phosphorylation induced by IGF-1. Pretreatment with PMA attenuated the tyrosine phosphorylation of IRS-1 as we reported in our previous study [42], and Akt while enhancing the phosphorylation of CREB (Fig 6).

### Treatment with a tyrosine kinase inhibitor blocks IGF-1 receptor signaling, including the phosphorylation of Akt and CREB

**Figure 7:**
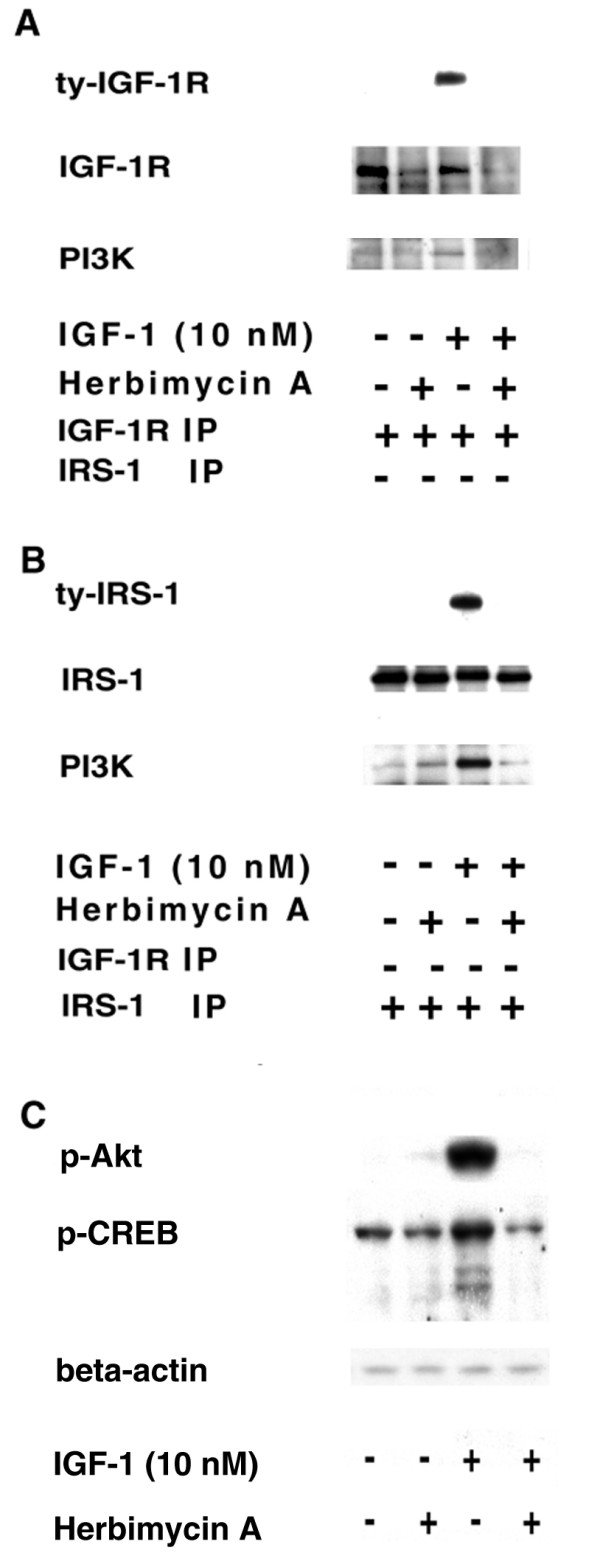
**The tyrosine kinase inhibitor herbimycin A blocked IGF-1 signalling, including Akt and CREB activation, in PC12 cells**. PC12 cells pretreated with or without herbimycin A were stimulated with 10 nM IGF-1 and the tyrosine phosphorylation of IGF-1 R (A) and IRS-1 (B), their association with PI3K, as well as the phosphorylation of Akt and CREB (C) were determined. IGF-1 stimulated the tyrosine phosphorylation of these markers. Pretreatment of PC12 cells with herbimycin A blocked these events. Blots represent prototypical example of experiments replicated at least 3 times.

Having established that IGF-1 stimulated the phosphorylation of Akt via a PI3 kinase pathway while that of CREB was mediated by the MAPK and p38 MAP kinase pathways, we studied next the upstream events involved in these effects. IGF-1 stimulated the tyrosine phosphorylation of IGF-1R (Fig 7A, lane 3 vs lane 1), IRS-1 (Fig 7B, lane 3 vs lane 1) and its association with PI3 kinase (Fig 7A and 7B, lane 3 vs lane 1), as well as those of Akt and CREB (Fig 7C lane 3 vs lane 1). Pretreatment with the tyrosine kinase inhibitor, herbimycin A, inhibited these actions of IGF-1 (Fig. 7A, 7B and 7C, lane 4 vs lane 3). Hence, the tyrosine phosphorylation of the IGF-1 receptor is essential for the activation/phosphorylation of these downstream signaling proteins.

### Both Akt and CREB can contribute to the survival effects of IGF-1 in PC12 cells

**Figure 8:**
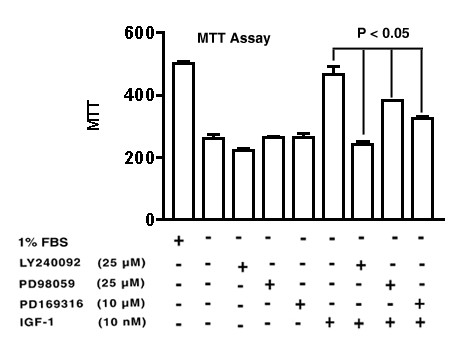
**Multiple signaling pathways are involved in the survival effect of IGF-1 in PC12 cells**. Following treatment with different kinase inhibitors for 40 min, PC12 cells were exposed to 10 nM IGF-1 for 24 hours and cell viability was determined by MTT as described in Methods. Cell survival of PC12 cells induced by IGF-1 is partially inhibited by various blockers, the PI3K inhibitor being the most effective. Data represent assays from at least three independent experiments.

Figure 8 shows that various kinase inhibitors for Akt, MAPK and p38 MAPK significantly attenuated the survival effects of IGF-1 with the PI3K/Akt kinase inhibitor being the most effective. Hence, while the PI3K/Akt pathway plays a major role in the protective effects of IGF-1, the MAPK/CREB pathway is also significantly involved even if to the lesser extent (Fig. 8).

### Effects of other trophic factors and agents in the phosphorylation of CREB

**Figure 9:**
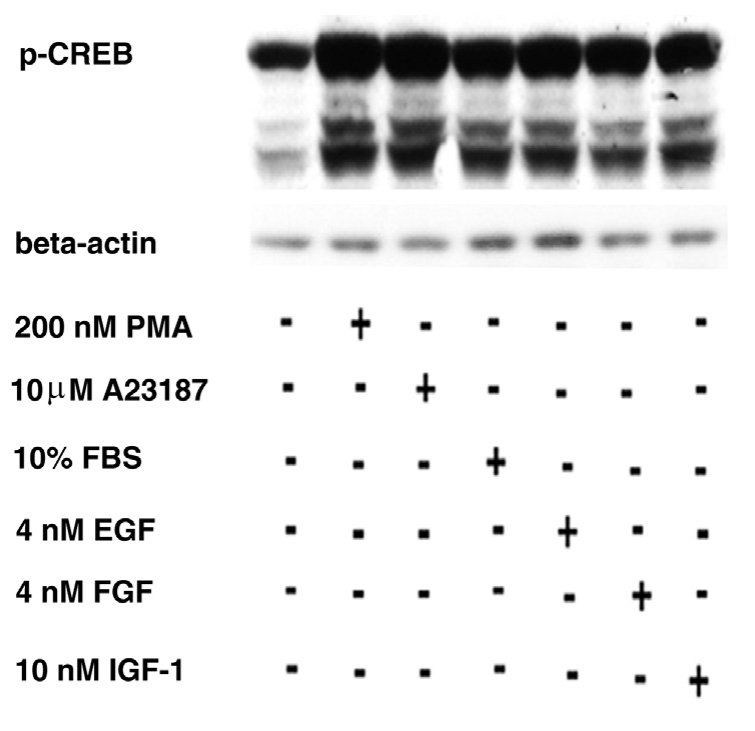
**EGF, FGF, FBS, PMA and the calcium ionophore-A23187 stimulated CREB phosphorylation in PC12 cells**. PC12 cells were treated with 10 nM IGF-1, 4 nM EGF, 4 nM FGF, 10% FBS, 200 nM PMA and 10 μM A23187 and the phosphorylation of CREB was determined as described in Methods. All these agents stimulated CREB phosphorylation in PC12 cells. Blots represent prototypical examples of experiments replicated at least 3 times.

To compare the effect of other factors and agents to that of IGF-1 on the phosphorylation of CREB, PC12 cells were treated with IGF-1, EGF, FGF, FBS, PMA and the calcium ionophore A23187, and the phosphorylation of CREB. Treatments with 4 nM EGF, 4 nM FGF, 10% FBS, 200 nM PMA and 10 μM A23187 significantly stimulated the phosphorylation of CREB that are comparable to the effect of IGF-1 (Fig 9).

## Discussion

The present study demonstrates that IGF-1 is able to time- and concentration-dependently stimulates the activation of both Akt and CREB in PC12 cells. The activation of Akt by IGF-1 is mediated by the PI3 kinase pathway while MAPK and p38 MAPK are involved in IGF-1 induced phosphorylation of CREB. Survival assay revealed that these various pathways contribute to the survival effects of IGF-1 in PC12 cells.

IGF-I is a polypeptide trophic factor capable of supporting growth and of preventing death in neuronal and non-neuronal cells. The biological functions of this growth factor are mediated by IGF-I receptors. Recent studies have shown that both IGF-1 and its receptors are expressed in the CNS [1,21,43], and their respective expression is upregulated in response to injuries [1,44]. It is also well established that IGF-1 protects the brain from hypoxic and ischemic injuries [45]. IGF-1 is also neuroprotective in a broad range of cells including cultured primary hippocampal neurons. These neuroprotective effects likely involve multiple signaling pathways but in particular the PI3K/Akt kinase and MAPK-CREB pathways [2,30,46,47]. Consistent with these findings, our results show here that IGF-1 is able to stimulate the activation of both the PI3K/Akt kinase and MAPK-CREB pathways in PC12 cells.

Although mechanisms underlying the phosphorylation of CREB have been extensively studied, the role of a given signaling pathway in mediating the effect of a trophic factor on CREB remains somewhat controversial. For example, while Akt has been suggested to act as a 'CREB kinase', data obtained here are not fully supportive of such an hypothesis. Indeed while IGF-1 induced the sustained phosphorylation of Akt, only a transient one was seen for CREB. Moreover, inhibitors of PI3K/Akt blocked the activation/phopsphorylation of Akt with almost no effect on the phosphorylation of CREB. In contrast, MAPK and p38 kinase inhibitors significantly diminished IGF-1-induced phosphorylation of CREB while only having a small effect on Akt. Indeed, the MAPK pathway inhibitor PD98059 and the p38 MAPK kinase inhibitor, PD169316, at concentrations fully inhibiting MAPK and p38 kinase respectively, significantly abrogated the phosphorylation of CREB while having no significant effect on the activation of Akt. Moreover, the phosphorylation of CREB induced by IGF-1 is not inhibited by Akt inhibitors at concentrations that fully blocked the phosphorylation of GSK3β, a target of Akt in the IGF-1 signaling pathway (data not shown). Finally, PMA, an activator of PKC, attenuated IGF-1-induced activation of Akt while enhancing the phosphorylation of CREB. Taken together, these data reveal that IGF-1-induced phosphorylation of Akt and CREB is mediated via distinct pathways and suggest that CREB is not a direct substrate of Akt in IGF-1 receptor signaling in PC12 cells.

In fact, MAPK and p38 MAPK most likely contribute to the phosphorylation of CREB stimulated by IGF-1 in PC12 cells. However, the existence of kinase(s) which can actually phosphorylate CREB at Ser-133 has not been reported in PC12 cells. MAPK and p38 MAPK cannot directly phosphorylate CREB at this residue as it is not a proline-directed phosphorylation site [41]. Downstream targets of these two kinases that may be able to phosphorylate CREB in PC12 cells include the ribosomal S6 kinase (RSK) family of kinases, MAPKAP kinase 2/3 and MSK1/2. For example, the over-expression of inhibitory RSK2 mutants reduced EGF-induced CREB phosphorylation [36] while cells deficient for this kinase were found to be resistant to EGF-stimulated CREB phosphorylation [48]. However, Rsk-2-deficient cell lines have shown that this kinase is not essential for the activation of CREB in response to PDGF and IGF-1 [49].

MAPKAP kinase-2, an enzyme immediately downstream of p38 MAP kinase, is able to phosphorylate CREB at Ser133 *in vitro*, and has been suggested to play an important role in FGF- or stress-induced phosphorylation of CREB and ATF-1 in SK-N-MC cells [34]. However, a subsequent report using PC12, HeLa and SK-N-MC cell lines failed to demonstrate a role for both RSK2 and MAPKAP-2/3 as CREB kinases in signalling induced by TNF, NGF and FGF [50]. The potential role of this kinase in IGF-1 induced CREB phosphorylation in PC12 cells hence remains to be fully established.

MSK1 is a downstream kinase of the MAPK and p38 MAPK kinase pathways and is important in stress and mitogen-induced CREB phosphorylation in fibroblasts, PC12 cells and embryonic stem cells [38,50-52]. Since both MAPK and p38 MAPK are involved in IGF-1 induced phosphorylation of CREB in PC12 cells, MSK1 is thus a possible intermediate step. Consistent with this hypothesis, preliminary data have shown that blockade of MSK1 significantly inhibited IGF-1 stimulated phosphorylation of CREB in PC12 cells (Zheng and Quirion, unpublished results).

The biological significance of the finding that distinct pathways are involved in IGF-1 induced phosphorylation of Akt and CREB is not clear. Both proteins are known to play central roles in cell survival [9,46,47,53]. The role of Akt in cell survival was proposed first by Dudek and colleagues (1997) in a study showing that IGF-1-induced survival of cultured cerebellar granule cells was mediated by this kinase [9]. Subsequently, Akt was shown to be a key survival promoting kinase for a broad of range of factors in a variety of cell type [1,22,46,47]. It is known that activated Akt can phosphorylate and hence inactivate proapoptotic proteins such as the Bcl-2 family member Bad [16], caspase-9 [17], GSK3 [15], FOXO transcription factors [21,22,47] and ASK1 [54]. Akt can also affect the expression of Bcl-2 family members in target cells as well as the function of NF-kappaB and CREB [46,47].

The MAPK-CREB pathway was also reported to play a major role in neuronal survival including in PC12 cells [47,53]. For example, MAPK can activate RSKs and MSK1/2 [51,53]. RSKs are then able to phosphorylate the pro-apoptotic protein Bad at Ser- 112suppressing Bad-mediated apoptosis [53]. RSKs and possibly MSK1/2 are also able to facilitate the phosphorylation of CREB at Ser-133, leading to cell survival. Accordingly, the Akt and MAPK-CREB pathways likely have additive effects in contributing to the survival effects induced by IGF-1 in PC12 cells. This is also exemplified by the action of Akt and MAPK in the phosphorylation of Bad. In the presence of growth factors, Akt is activated and phosphorylates Bad at Ser-136, inhibiting its pro-apoptotic effect [16]. In parallel, the activation of MAPK leads to the phosphorylation of Ser-112 further inhibiting Bad [53]. Hence, IGF-1 by acting via the IGF-1 receptor complex and parallel downstream effectors can inhibit various pro-apoptotic signals in a variety of cells including PC12 cells.

## Conclusion

In conclusion, this study shows that IGF-1-induced phosphorylation of Akt kinase is mediated by the PI3 kinase pathway while that of CREB is regulated mainly by activating the MAPK and p38 MAPK pathways.

## Methods

### Materials

Human recombinant IGF-1 was obtained as a gift from Genentech Inc (San Francisco, CA). LY294002, PD98059, herbimycin A, rapamycin, PD169316, Akt inhibitors I and II were purchased from Calbiochem (Bad Soden, Germany) whereas wortmannin, leupeptin, aprotinin, sodium vanadate and phorbol 12-myristate 13-acetate (PMA) were from Sigma Chemical (St Louis, MO). U0126 was purchased from Promega (Madison, WI). Anti-phospho-CREB-133, anti-CREB, anti-phospho-p38 MAPK, anti-phospho-Akt, anti-phopho-GSK3β (Ser-9) and anti-phospho-MAPK antibodies were obtained from Cell Signaling Technology (Beverly, MA). Anti-Akt, anti-MAPK, anti-p38 MAPK and all secondary antibodies conjugated with horseradish peroxidase (HRP) were from Santa Cruz Biotechnology (Santa Cruz, CA). Cell culture reagents were purchased from GIBCO Life Technologies (Grand Island, NY) whereas all other reagents were from Sigma Chemical or Fisher Scientific (Nepean, ON).

### Cell culture

PC12 cells were kindly provided by Dr. Gordon Guroff at NICHD, NIH (Betheda, MD) and cultured as described before. In brief, PC12 cells were maintained in 75 cm2 flasks in high glucose Dulbecco's Modified Eagle's Medium (DMEM) supplemented with 5% (vol/vol) fetal bovine serum (FBS), 5% horse serum, 100 μg streptomycin/ml and 100 U penicillin/ml. Cells were incubated at 37°C with 5% CO2 humidified atmosphere. Stock culture was routinely sub-cultured at 1:5 ratio at a week interval.

### Treatments

Before each experiment, cells were detached using 5 mM EDTA in Hank's balance buffer (HBS) and seeded in 12 or 6 well plates (coated with poly-D-lysine, 10 μg/ml) at a density of 3–6 × 10^5^cells/well in 2% serum medium for 24 hrs. Culture medium was replaced with DMEM 2 hr before the desired reagents were added. To study the effect of IGF-1 on the phosphorylation of Akt, CREB, MAPK, p38 MAPK and GSK3beta, cells were treated with 10 nM IGF-1 for 10 min. Alternatively, cells were pretreated with wortmannin (0–2 μM, 30 min), LY294002 (6–50 μM, 30 min), rapamycin (50 nM, 30 min), PD98059 (6–50 μM, 40 min) or PD169316 (2.5–20 μM, 40 min) followed by a stimulation with 10 nM IGF-1. For the experiments with PKC, 400 nM PMA was added to cells 2.5 min prior to IGF-1 stimulation.

### Western blotting

Western blotting was performed as described earlier with some modifications [1,23]. Briefly, treated cells from different experimental conditions were rinsed twice with ice cold HBSS and lysed in either sample buffer [62.5 mM Tris-HCl, pH 6.8, 2% w/v sodium dodecyl sulfate (SDS), 10% glycerol, 50 mM dithiothreitol (DTT) and 0.1% w/v bromphenol blue] or RIPA buffer [50 mM Tris-HCl, pH 8.0, 150 mM NaCl, 1 mM EDTA, 1% Igepal CA-630, 0.1% SDS, 50 mM NaF, 1 mM NaVO3, 5 mM phenyl-methylsulfonyl fluoride, 10 μg/ml leupeptin, 10 μg/ml aprotinin]. Samples with equal amounts of protein were then separated by 4–20% polyacrylamide gel electrophoresis (PAGE), and the resolved proteins were electrotransferred to Hybond-C Nitrocellulose. Membranes were incubated with 5% non-fat milk in TBST (10 mM Tris-HCl, pH 8.0, 150 mM NaCl and 0.2% Tween-20) for 1 hr at room temperature and incubated with appropriate primary antibody at 4°C overnight. Membranes were then washed twice with TBST and probed with corresponding second antibodies conjugated with HRP (anti/rat/mouse/rabbit/goat-HRP) at room temperature for 1 hr. Membranes were finally washed several times with TBST to remove unbound secondary antibodies and visualized using an ECL detection kit (Amersham Co, Toronto, ON). A part of the SDS gel was stained with Coomassie Blue to ensure the use of equal amounts of protein.

The respective phosphorylation of Akt, MAPK, CREB, GSK3β (Ser-9) and p38 MAPK was determined by Western blot using anti-phospho-Akt, anti-phospho-MAPK, anti-phospho-CREB, anti-phopho-GSK3β (Ser-9) and anti-phospho-p38 MAPK antibodies, respectively. Blots were stripped and reprobed with anti-Akt, anti-MAPK, CREB, GSK3β and p38 MAPK antibodies to ensure that equal amounts of various proteins were present. The effect of IGF-1 was determined by comparing the phosphorylation of above protein and their unphosphorylated counterpart or beta-actin levels in cell extracts determined as mentioned above.

### Determination of tyrosine phosphorylation of the IGF-1 receptor, IRS-1 and their interactions with PI3 kinase was established by immunoprecipitation

PC12 cells were treated with 10 nM IGF-1 for 8 min and rinsed with cold phosphate buffer saline (PBS). After centrifugation at 1000 g for 5 min at 4°C, cell pellets were lysed on ice in pre-cold RIPA buffer for 20 min. Cell lysates were then pelleted at 13,000 g for 10 min and the concentration of protein in each sample was determined using the Bio-Red dye-binding method with bovine serum albumin (BSA) as standard. The supernatant with equal amount of protein was incubated overnight at 4°C with either anti-IGF-1R, anti-IRS-1 or anti-PI3 kinase antibodies. Formed immunocomplexes were isolated by protein A/G PLUS-agarose (Santa Cruz Biotechnology, Inc.), separated by 4–20% SDS gel and then tyrosine phosphorylation was determined by Western blot with a mixture of anti-phosphotyrosine antibodies 4G10 and PY99. Blots were striped and reprobed with PI3 kinase or IRS-1 antibodies to evaluate the interaction of IGF-1R and IRS-1 with PI3 kinase. Finally, the blots were reprobed with anti-IGF-1R, anti-IRS-1 or anti-PI3 kinase to ensure the presence of equal amount of proteins.

### Cell viability using the MTT assay

PC12 cells (10000 to 20000 cells/well) in serum -free medium DMEM or DMEM supplemented with 1 % FBS were added to 96- well plates and incubated at 37°C with 5% CO2 for 1 h. Cells were pretreated with 25 μM LY294002, 25 μM PD98059, 10 μM PD169316 for 40 min and then 1 % FBS and 10 nM IGF-1 for 24–48 hours. Following replacement of the medium with 0.5 mg/ml MTT in DMEM, cells were returned into the incubator for a 3-hr period. Cells and MTT formazan crystals were then solubilized by trituration in a solution of isopropanol/HCL (0.1 N) and the survival profile of these cells were quantified by spectrophotometrically measuring the plate at 570 nM. Assays were repeated at least three to six times, each in quadruplicate.

### Statistical analysis

Data are expressed as mean ± SEM. A one-way ANOVA with Newman-Keul test was used to establish statistical significance set at p < 0.05.

## Authors' contributions

WHZ did experiments, collected data and prepared manuscript. RQ contributed in manuscript preparation.
